# Distinctive pattern of neutrophil count change in clozapine-associated, life-threatening agranulocytosis

**DOI:** 10.1038/s41537-022-00232-0

**Published:** 2022-03-14

**Authors:** David Taylor, Kalliopi Vallianatou, Eromona Whiskey, Olubanke Dzahini, James MacCabe

**Affiliations:** 1 Institute of Pharmaceutical Science, King’s College London, Franklin-Wilkins Building, 150 Stamford Street, London, SE1 9NH UK; 2 Pharmacy Department, Maudsley Hospital, Denmark Hill, London, SE5 8AZ UK; 3 National Psychosis Unit, Bethlem Royal Hospital, Monks Orchard Road, Beckenham, Kent BR3 3BX UK; 4 Department of Psychosis Studies, Institute of Psychiatry Psychology and Neuroscience, King’s College London, De Crespigny Park, London, SE5 8AF UK

**Keywords:** Schizophrenia, Psychiatric disorders

## Abstract

The wider use of clozapine is limited by the risk of agranulocytosis and the associated requirement for monitoring of neutrophil counts. We searched local electronic patient records for cases of agranulocytosis occurring during clozapine treatment during the period 2007–2020. We found 23 episodes recorded as agranulocytosis in clozapine patients. Of these, nine met pre-defined criteria and were considered episodes of life-threatening agranulocytosis (LTA). These episodes of clozapine-induced LTA exhibited a distinct pattern of continuous and rapid neutrophil count decline to zero or near zero. Mean time for neutrophils to fall from ANC > 2 to ANC <0.5 × 10^9^/L was 8.4 days (range 2–15 days). Each event was also characterised by a prolonged nadir and delayed recovery (range 4–16 days). Non-LTA episodes were, in contrast, brief and benign. We conclude that an important proportion of cases of agranulocytosis identified in people prescribed clozapine are not life-threatening and may not even be clozapine-related. Monitoring schemes should aim to identify true clozapine-induced LTA as opposed to threshold-defined nominal agranulocytosis. Genetics studies might benefit from examining associations with clozapine-induced LTA rather than with recorded cases of agranulocytosis or neutropenia.

## Introduction

Clozapine was the first drug found to possess antipsychotic action without causing extrapyramidal effects^1^. It eventually gained product registration and was marketed in Europe the early 1970s. Clozapine’s association with agranulocytosis was first reported in 1975 when Finnish authorities described eight cases occurring in around 2000 patients prescribed clozapine^2^ (an incidence of approximately 0.4%). Outside Finland, the risk of clozapine-associated agranulocytosis at that time was estimated to be 0.027%^3^. A later and more complete analysis of the cases occurring in Finland suggested that there were “17 cases of neutropenia or agranulocytosis amongst about 3000 patients treated”^4^. In fact, there had been ten cases of agranulocytosis (seven of which were fatal)—an incidence of agranulocytosis of 0.33%. Clozapine was withdrawn worldwide and later reintroduced with strict haematological monitoring.

The initial association detected was with fatal or near fatal agranulocytosis. However, the introduction of mandatory haematological monitoring for clozapine suggested that clozapine was associated with both agranulocytosis (defined, in respect to clozapine use, as neutrophils <0.5 × 10^9^/L) and neutropenia (usually defined as a neutrophil count of <1.5 × 10^9^/L; sometimes <2 × 10^9^/L). In patients registered on formal clozapine monitoring schemes, the incidence of neutropenia has been reported to range from 2.5^5^ to 5.5%^6^ and the incidence of agranulocytosis from 0%^7,8^ to as much as 1%^6,9^. Clozapine remains underutilised because of the fear of both neutropenia and agranulocytosis and because of the burden imposed by regular blood testing^10,11^.

Clozapine’s association with neutropenia is rarely questioned but clozapine may not in fact cause a benign neutropenia. In the original Finnish analysis^4^ the incidence of neutropenia that did not progress to agranulocytosis was 7 in 3000, or 0.23%—less than a tenth of later estimates of the incidence of neutropenia. It is possible that the perceived association with neutropenia is a result of surveillance bias, whereby intensive blood monitoring reveals random, clinically silent, and non-pathological episodes of neutropenia occurring coincidentally to the use of clozapine. This is important because a great many people are prevented from receiving clozapine because of erroneously diagnosed clozapine-related blood toxicity^12^. The concept of clozapine-induced neutropenia might also be a blind-alley for research into genetic predisposition to clozapine-induced blood dyscrasia. Although the same genetic variants seem to be responsible for the increased risk of both neutropenia and agranulocytosis^13^, a recent study found a strong link between the human leucocyte antigen (HLA) polymorphism rs113332494 and agranulocytosis but not neutropenia^14^. Moreover, in studies where genetic associations with clozapine-associated neutropenia are suggested^15^, it is acknowledged that the majority of neutropenia cases do not develop into agranulocytosis.

It could be argued that what really matters in respect to the safe use of clozapine is the detection and treatment of episodes of reduced neutrophil count that are sufficiently severe and prolonged to represent a risk to life. Few studies have investigated the time course of neutrophil count before, during and after confirmed cases of clozapine-induced life-threatening agranulocytosis (LTA). The aim of this study was to explore the pattern of neutrophil count in patients with true clozapine-induced LTA and to characterise this reaction so as to distinguish it from benign and coincidental episodes of neutropenia. Our hypothesis was that true clozapine toxicity might be distinguished from benign events by carefully describing the pattern of neutrophil count changes in life-threatening cases.

## Results

### Case selection

**Fig. 1:**
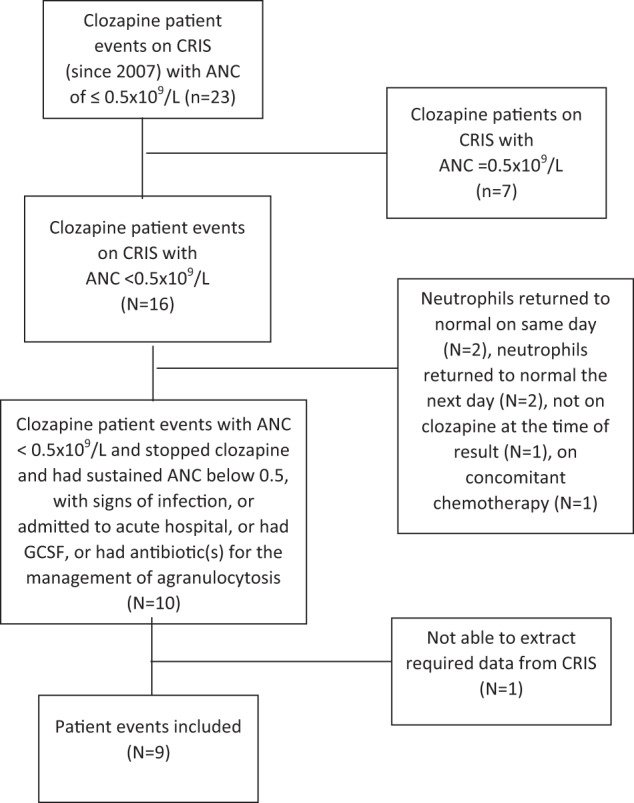
Case selection.

Figure 1 shows a flow diagram of the final case selection. We identified 23 events in which ANC fell to 0.5 × 10^9^/L, 16 of these in which an ANC below 0.5 × 10^9^/L was recorded. Seven of these sixteen cases were excluded; four because their neutrophil count returned to normal on the same day or on the next day without there being two consecutive ANCs below 1.0 × 10^9^, one because the patient had stopped clozapine and switched to quetiapine before neutrophils began to decline, one because they were on concomitant cancer chemotherapy at the time of the event and one because we could not access any ZTAS blood test results via CRIS (and therefore had an incomplete record of ANC results). The study cohort included nine events in eight patients.

Table 1 illustrates the demographic and clinical characteristics of cases.

**Table 1:** Demographic and clinical characteristics of study cohort.

Male	(67%)
Ethnicity	
White British/white other	6
Asian/Asian British Pakistani	2
Black/Black British Caribbean	1
Diagnosis	
F20 Schizophrenia	8
F25 Schizoaffective disorder	1
Mean age at initiation of treatment (years)	48.6
Median age (min, max) (years)	51 (25,72)
Comorbidities (*n*)	
Hypothyroidism	2
Multiple comorbidities	1
Concurrent medication	
Lithium	2
Valproate	1
Lamotrigine	1
2nd antipsychotic	1
Ramipril	1
BEN criteria used	2
BEN criteria AND lithium	1
Tobacco smokers	7
Frequency of FBC monitoring	
Weekly	9
Two-weekly	0
Four-weekly	0
Mean clozapine dose (SD) mg/day at *t* = 0	300 (84.5)
Median clozapine dose (Min, Max) mg/day at *t* = 0	275 (200, 450)
Mean duration of clozapine treatment up to *t* = 0 (days) (range)	48 (21–105)
Days to recover ANC > 1.5 (not given G-CSF)	
Mean (SD)	13 (4.2)
Median (min, max)	13 (10,16)
Days to recover ANC > 1.5 (given G-CSF)	
Mean (SD)	10.7 (2.8)
Median (min, max)	10 (4,12)
Number of events with GCSF given	7
Number of cases hospitalised	6
Number of cases received	
Antibiotics	6
Antivirals	1

The temporal profiles of the absolute neutrophil counts for the nine subjects identified are depicted in the graphs appended (Figs. 2–10 (in each graph the *y*-axis represents neutrophil count (0–15 × 10^9^/L) and the *x*-axis 38 days before and 38 days after day 0 (the day on which a count below 0.5 × 10^9^/L was first recorded in each case)).

**Fig. 2:**
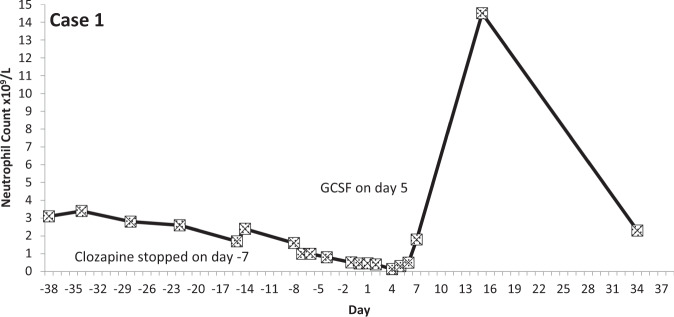
Neutrophil count pattern for Case 1.

**Fig. 3:**
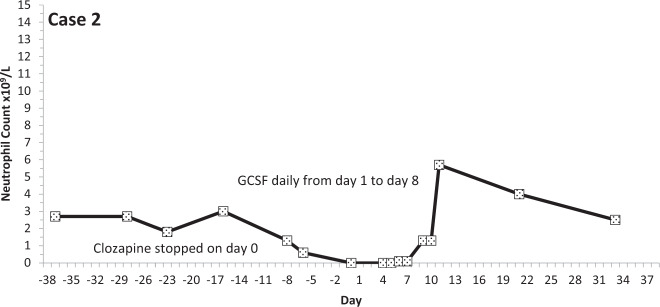
Neutrophil count pattern for Case 2.

**Fig. 4:**
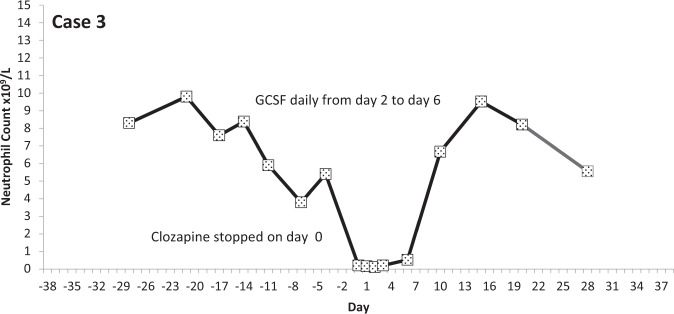
Neutrophil count pattern for Case 3.

**Fig. 5:**
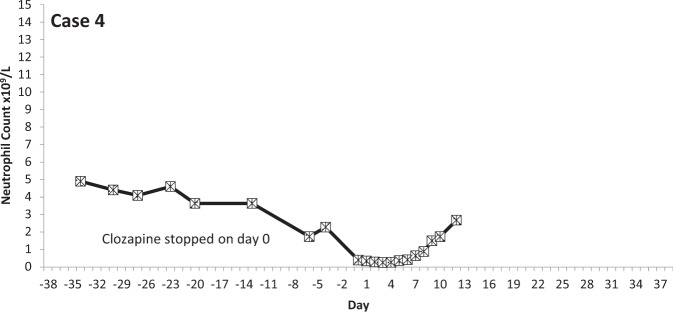
Neutrophil count pattern for Case 4.

**Fig. 6:**
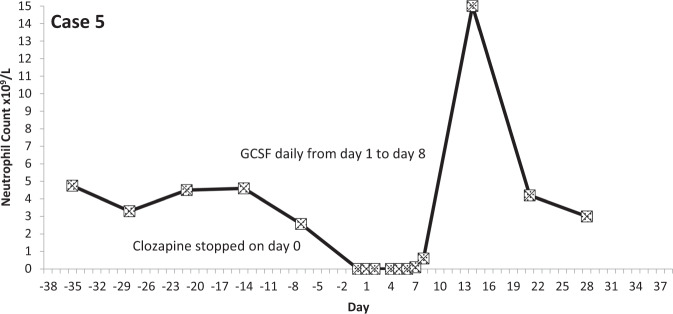
Neutrophil count pattern for Case 5.

**Fig. 7:**
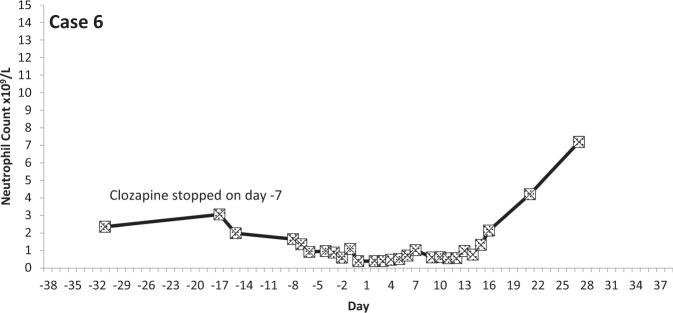
Neutrophil count pattern for Case 6.

**Fig. 8:**
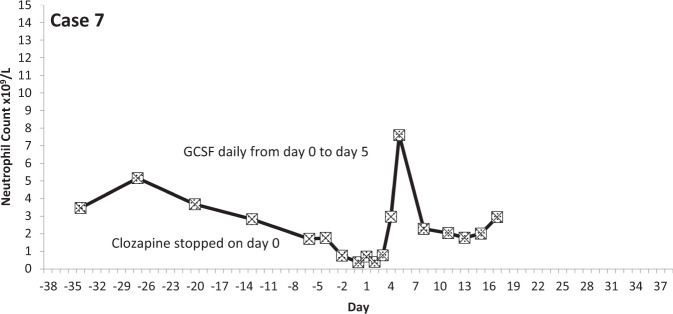
Neutrophil count pattern for Case 7.

**Fig. 9:**
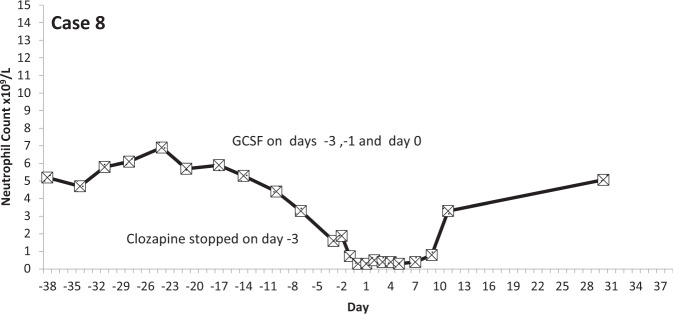
Neutrophil count pattern for Case 8.

**Fig. 10:**
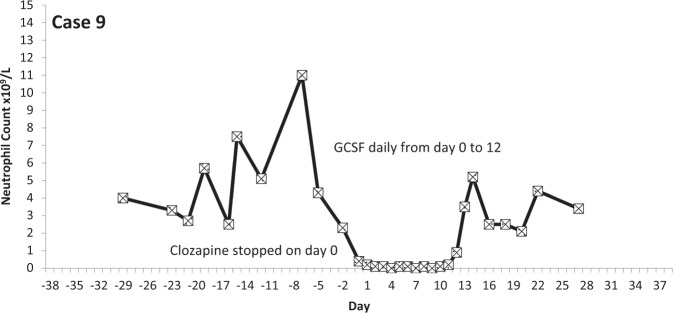
Neutrophil count pattern for Case 9.

### Case details

There were nine events in eight patients who had a sustained neutrophil count <0.5 × 10^9^/L, stopped clozapine and either were hospitalised and/or received GCSF and/or antibiotics (Table 2). Seven of the cases had been prescribed clozapine on a previous occasion. In six of these cases clozapine was previously discontinued owing to neutropenia. None had previously had agranulocytosis (with the exception of the patient contributing two “cases” in this study): the lowest ANC recorded for any patient in previous exposures was 0.8 × 10^9^/L. Two cases were in patients having their first trial of clozapine.

**Table 2:** Details of LTA cases.

Patient ID code	Number of previous episodes of clozapine use	Registered BEN status	Prescribed lithium	Time from clozapine start to ANC < 0.5 (days)	Time from last count of ANC > 2 to first count of ANC < 0.5 (days)	Signs of infection	Rx antibiotics	Admitted to acute hospital	“Agranulo-cytosis” recorded in clinical notes	GCSF prescribed and given
Subject 1	1	No	No	44	14	Yes	Yes	Yes	Yes	Yes
Subject 2	0	Yes	No	105	10	Yes	Yes	Yes	Yes	Yes
Subject 3	2	No	No	21	4	Yes	Yes	Yes	Yes	Yes
Subject 4	1	Yes	Yes	36	4	No	Yes	No	No	No
Subject 5	2	No	No	34	7	Yes	Yes	Yes	Yes	Yes
Subject 6	1	No	No	63	15	No	Yes	Yes	No	No
Subject 7	0	No	No	61	13	Yes	No	No	Yes	Yes
Subject 8	2	No	Yes	42	7	No	No	No	No	Yes
Subject 9	2	No	No	26	2	Yes	Yes	Yes	Yes	Yes

One patient had stopped clozapine previously owing to poor compliance and, upon re-starting treatment, had developed LTA. Following a further break of several months, an attempt to re-challenge with clozapine again resulted in LTA. This patient thus provides two “cases” of LTA.

Six of nine patients required transfer to an acute hospital for close monitoring. Five of these had IV antibiotics for treatment of infection and one had an antiviral plus an antifungal prescribed. Three cases remained on the psychiatric hospital premises, one was administered antibiotics but not GCSF, two had GCSF but not antibiotics. Overall, GCSF was used in seven out of nine events.

All events occurred within the first 18 weeks of starting treatment. In five of the nine events of LTA, the neutrophil count was reduced from above two to less than 0.5 × 10^9^/L within 7 days (the blood testing interval for these patients). The mean time for neutrophils to fall from ANC > 2 to ANC <0.5 × 10^9^/L was 8.4 days (range 2–15 days; median 7 days).

Note: regulations relating to the identification of patients on the CRIS database preclude specific reference to individual details, so as to preserve anonymity.

## Discussion

We defined clozapine-associated LTA as that occurring in people stopping clozapine when two consecutive neutrophil counts were recorded as being below 0.5 × 10^9^/L/1.0 × 10^9^/L and where there was evidence of infection, or the prescribing of antibiotics or G-CSF. The nine cases we identified showed a consistent pattern: neurophil counts fell quickly from a normal level and remained substantially below 0.5 × 10^9^/L for several days until natural recovery or until GCSF took effect.

A previous study examined the time course of neutrophil counts in 38 patients stopping clozapine owing to agranulocytosis (defined as ANC <0.5 × 10^9^/L) and found that neutrophil counts fell from above 2.0 × 10^9^/L to below 0.5 × 10^9^/L in an average (mean) of 1 week^16^. The median time to obtain counts above 0.5 × 10^9^/L on recovery was 4 days and to achieve normal counts, 10 days. All of these observations are remarkably similar to the findings in this study. Further, published individual case studies describe a near identical pattern of neutrophil count change in LTA^17,18^. Some authors have reported that neutrophil counts increase immediately before falling to levels representing agranulocytosis^19^ but this phenomenon was evident in only two, perhaps three, of our nine cases.

Clozapine-induced agranulocytosis is a rare event^20^ and its pathogenesis is not fully understood^21^. The formation of reactive clozapine metabolites may be directly toxic to neutrophils^22^. Studies also suggest immunomodulatory effects resulting in transient changes in neutrophil counts after treatment initiation^23^. Spikes in WCC and neutrophils from the first week of treatment, stabilising by week 10 have been reported^20,24^. Re-challenge may result in rapid recurrence and more dramatic dyscrasia^25^, an observation which supports an immune-mediated reaction. Genetic association with clozapine-induced agranulocytosis suggests immune mechanism involving human leucocyte antigen genes^13^.

Distinct mechanisms and risk factors may underlie clozapine-associated benign neutropenia and clozapine-induced agranulocytosis^26^. Based on incidence figures, at least two thirds of cases of neutropenia identified in people on clozapine do not “progress” to agranulocytosis. As already mentioned, these cases of neutropenia may be neither clozapine-related nor pathogenic and so may have no mechanism to speak of. In a US general population study^27^ the prevalence of neutropenia was 4.5% in black participants and 0.78% in white participants. These cases appear to be naturally occurring episodes of low neutrophil counts that may have no clinical significance. The repeated blood testing required for clozapine use undoubtedly increases the chances of finding cases of neutropenia, which may then be inappropriately linked to the use of clozapine. A meta-analysis of twenty studies reporting rates of neutropenia with clozapine and an active comparator, found no elevated risk for clozapine^28^. In a more recent study in Iceland^29^ (where clozapine blood monitoring is less frequent than elsewhere), the incidence of neutropenia was no greater with clozapine than with other antipsychotics and, of 34 neutropenia cases identified in people on clozapine, only one progressed to LTA.

Even cases of agranulocytosis in people on clozapine may be either benign and conicidental cases or clozapine-associated LTA. We excluded 14 patients who would normally be diagnosed as having clozapine-induced agranulocytosis (ANC ≤ 0.5 × 10^9^/L). In most of these cases neutrophils rapidly returned to normal with no sequelae. This is not the first time that this phenomenon has been reported—in 1992 Hummer and colleagues^30^ described transient neutropenia occurring in 22% of patients prescribed clozapine. Two of these patients (2.9% of the total cohort) briefly recorded counts below 1.0 × 10^9^/L. In the Icelandic study mentioned^29^ several patients recorded ANC results that in other countries would have forced clozapine cessation, but almost all continued without any problems, Interestingly, in the population prevalence study already cited^27^, neutrophil counts below 1.0 × 10^9^/L were recorded in 0.57% of black patients and 0.11% of white participants. Another population analysis^31^ suggested the incidence of agranulocytosis (ANC ≤ 0.5 × 10^9^/L) casually detected in people not receiving cancer chemotherapy or immunosuppressant drugs is 10.4/million/year for black people and 4.4/million/year for whites. Thus, there is a natural background of very low neutrophil counts, the incidence of which is fully exposed by the intensive monitoring seen with clozapine.

Our study was not designed to estimate the incidence of LTA and we have no precise figures on clozapine usage over the time period examined. An approximate estimate can be made, however. Our unit has around 1400 patients currently prescribed clozapine and each year around 250 new patients are initiated on clozapine. So, over the 14 years of this study approximately 3500 patients were started on clozapine and there were 23 recorded cases of “agranulocytosis”, giving an incidence of 0.66%—a figure within the range reported by larger and dedicated studies. Note however that the incidence of LTA (nine cases in approximately 3500 starters) was only 0.26%. This illustrates a further important aspect of our findings: that the incidence of LTA is probably substantially less than the accepted risk of agranulocytosis in people taking clozapine.

These observations have considerable importance when we consider rules relating to the use of clozpine. The UK haematological monitoring guidelines for patients on clozapine recommend a weekly ANC and WCC for the first 18 weeks, 2-weekly for weeks 19 to 52 and 4-weekly after 1 year of ongoing treatment with stable blood counts^32^. Clozapine treatment is discontinued if the WCC drops <3 × 10^9^/L and/or neutrophil count <1.5 × 10^9^/L, which is termed a “red” result and treatment must be stopped. For patients with benign ethnic neutropenia (BEN) this threshold is lower by 0.5, and treatment is discontinued when WCC drops <2.5 × 10^9^/L or ANC <1 × 10^9^/L. Following two “red” results the patient is classed as non-re-challengeable^32^ and may not receive clozapine again. In the US the threshold for clozapine treatment interruption was reduced to <1 × 10^9^/L in 2015. For patients with BEN this threshold was reduced to ANC <0.5 × 10^9^/L^33^. The US changes simplify clozapine monitoring and enable more patients to initiate and continue treatment without endangering their health^34^. A lower threshold of 0.5 × 10^9^/L also is the most cost-effective method of blood monitoring with clozapine^35^, in the absence of genetic testing. Alignment of the UK guidelines with the revised US Food and Drug Administration monitoring guidelines would have a similar positive impact on improving the use of clozapine without compromising safety^12,36^. Notably, nonetheless, although sequential falls in ANC may be reported to prescribers, both US and UK regulations classify clozapine-induced blood dyscrasia somewhat crudely according to predefined threshold counts, rather than the identification of a pathological pattern of decline in neutrophils that clearly indicates clozapine as the cause.

As already stated, it is highly likely that standard monitoring thresholds overestimate the proportion of patients who have true clozapine-related LTA. Indeed, it has been shown that the majority of those on the UK non-rechallenge database can be safely re-prescribed clozapine^12^. A substantial fraction of apparent clozapine-related neutropenia (and even agranulocytosis) is actually a manifestation of benign neutropenia, in which successful rechallenge is possible^37^.

There are two major consequences to this overdiagnosis. First, many people are unnecessarily precluded from receiving clozapine because of an assumed clozapine-related blood toxicity. Second, genetic association studies are compromised by the inclusion of cases of neutropenia and agranulocytosis that are neither related to clozapine nor of clinical consequence. The highest sensitivity value ever obtained for a genetic test for clozapine-related agranulocytosis is 54%^14^. However, all studies so far conducted defined agranulocytosis as any measurement of ANC ≤ 0.5 × 10^9^/L and so inevitably included in their study samples a proportion of people with a nominal and inconsequential agranulocytosis. Higher sensitivity values are unlikely to be attained without refining case-selection in line with the findings presented here.

Our results and other cases reported in the literature indicate that clozapine-associated LTA is an all-or-nothing event—a rapidly emerging absence or near absence of circulating neutrophils caused by their almost total chemical or immunological destruction or by the disabling of all neutrophil production by some means (the biological half-life of neutrophils in the blood is 7–9 h^38^). LTA associated with clozapine has a distinct pattern, which involves a precipitous and continuous fall in neutrophil counts to zero or near zero and their slow recovery after several days, with or without GCSF. The speed at which LTA develops is such that monitoring at intervals longer than 1 or 2 weeks is unlikely to help identify cases. Indeed, evidence is now emerging that longer-than-normal monitoring intervals employed during the COVID-19 pandemic may not have led to adverse consequences^39^. We further contend that single episodes of reductions in neutrophil counts—even at or those below 0.5 × 10^9^/L officially registering as agranulocytosis—are highly unlikely to be clozapine-related or life-threatening. Likewise, it does not seem likely that clozapine causes benign, non-progressive neutropenia. These episodes can be seen as being an artefact of the intensive blood monitoring programme, which exposes naturally occurring low neutrophils counts which are likely to be seen in any population.

We conclude that clozapine-related blood toxicity should be identified on the basis of the temporal pattern of change of neutrophil counts, not by the attainment of an arbitrary threshold neutrophil count. The value of blood monitoring at intervals of four weeks should be reconsidered because of the low probability of detecting true clozapine-related agranulocytosis (which develops over a period of 2 weeks or less).

## Methods

We used the Clinical Record Interactive Search (CRIS) system at the South London and the South London and Maudsley NHS Foundation Trust Biomedical Research Centre (BRC) to extract clinical data. CRIS is a research database that draws anonymised data from the electronic health records of patients at the South London and the Maudsley NHS Foundation Trust (SLAM), a mental health service that serves over 1.3 million people in four London Boroughs^40^. This research repository allows real-time searches of anonymised free text and structured data of SLAM electronic health records from 2007 onwards. The search results are returned as spreadsheets and exported as CSV files for subsequent analysis. There is data linkage between CRIS and the Zaponex Clozapine Monitoring System (ZTAS) used in SLAM, enabling access to blood test results from this register^40,41^.

We searched for, and assessed for inclusion, data on all patients who discontinued clozapine owing to a recorded neutrophil count of 0.5 × 10^9^/L or lower (the usual definition of agranulocytosis) over the time period 2007 to the end of 2020.

### Selection of LTA cases

We began by conducting an automated search which returned all clozapine patients with a neutrophil event ≤0.5 × 10^9^/L on CRIS. We then excluded all events where patients had an ANC exactly equal to 0.5 × 10^9^/L, and whose count did not fall below this threshold throughout the monitoring period. Further information on signs of infection (e.g., fever/raised CRP), use of GCS-F, transfer to acute hospital, administration of antibiotics during the event and advice sought from a haematologist were extracted by manual search of the anonymised case notes on CRIS. We defined as time zero (*t* = 0) the day that the neutrophil count was first found to be below 0.5 × 10^9^/L. Under the UK clozapine monitoring rules, patients must discontinue clozapine when their neutrophil count falls below 1.5 × 10^9^/L on two consecutive days.

**The LTA inclusion criteria were:**

- A recorded neutrophil count of less than 0.5 × 10^9^/L in patients who stopped clozapine because of this low count.
- Signs of infection (raised temperature and/or raised CRP) and/or admitted to general hospital for the treatment of infection and/or prescribed antibiotics and/or G-CSF administered for the management of agranulocytosis.

Included patients must have recorded at least two consecutive ANC of less than 1.0 × 10^9^/L (i.e., the first ANC <0.5 × 10^9^/L (as above), and the next ANC <1.0 × 10^9^/L). In this way we excluded those with transient low counts. We also excluded patients on clozapine and concomitant cancer chemotherapy.

### Data extraction

Neutrophil counts before and after *t* = 0 were extracted from laboratory results and from a manual search of the anonymised free text entries on CRIS. Demographic information on gender, ethnicity, diagnosis was also collected from CRIS. The clozapine starting date, the patient’s age at the time of the event (*t* = 0), concomitant medication, comorbidities, smoking status, frequency of blood count monitoring, as well as whether clozapine treatment was prescribed under the specific protocol Benign Ethnic Neutropenia, were extracted using a structured search. We identified whether the patient had a history of prior clozapine prescription and if clozapine had previously been stopped as a result of clozapine-related neutropenia or agranulocytosis.

### Data analysis

The mean, standard deviation (SD) and median values of variables were calculated for descriptive statistics. We used Excel Version 2107 to categorise the data and plot temporal profiles of each subject’s ANC for visualisation.

## Data Availability

Anonymised data are available from the corresponding author.
